# Context modulates the impact of auditory information on visual anticipation

**DOI:** 10.1186/s41235-022-00425-2

**Published:** 2022-08-02

**Authors:** Rouwen Cañal-Bruland, Hauke S. Meyerhoff, Florian Müller

**Affiliations:** 1 Department for the Psychology of Human Movement and Sport, Institute of Sport Science, Friedrich Schiller University Jena, Seidelstraße 20, 07749 Jena, Germany; 2 University of Erfurt, Erfurt, Germany; 3 Leibniz-Institut Für Wissensmedien, Tübingen, Germany

**Keywords:** Context, Auditory perception, Multisensory integration, Anticipation, Sport

## Abstract

Research on the impact of auditory information on visual anticipation in tennis suggests that the intensity of racket-ball-contact sounds systematically biases estimates of the ball’s speed, thereby influencing anticipatory judgments. Here we examined whether the effect of auditory information on visual anticipation is dependent on the sport-specific context in two separate experiments. In Exp. 1, participants watched short videos of tennis rallies that were occluded at racket-ball-contact. Racket-ball-contact sounds of the final shot were either present or absent. Participants faced different tasks in two counterbalanced blocks: In one block they estimated the ball’s speed; in the other block they indicated the ball’s landing location. Results showed that participants estimated longer ball flight trajectories and higher ball speeds in the sound present condition than in the sound absent condition. To probe whether this effect is dependent on the sport-specific context, Exp. 2 introduced an abstract (i.e., context-free) version of the previous stimuli. Based on the ball locations in the original videos used in Exp. 1, we rendered new videos that displayed only a moving circle against a blank background. Sine tones replaced the original racket-ball contact sounds. Results showed no impact of sound presence on location anticipation judgments. However, similar to Exp. 1, object speeds were judged to be faster when the final sound was present. Together, these findings suggest that the impact of auditory information on anticipation does not seem to be driven by sound alone, but to be moderated by contextual information.

## Significance statement

Due to the extreme time constraints in fastball sports such as tennis, anticipating the outcome of an opponent’s action is a prerequisite for successful performance. Recent research on anticipation in sport shows that besides visual information, observers integrate and use auditory cues. For instance, in tennis sounds emanating from racket-ball-contact serve to estimate how hard a ball is struck and predict its landing position on the court. At the same time, recent basic research on audio-visual integration suggests that the synchronous presentation of an audio (i.e., beep tone) and a visual (i.e., an object changing direction) stimulus may cause an illusory increase in perceived speed, raising the question whether the effects of auditory information on anticipatory judgments may represent a perceptual phenomenon that is independent of the sport-specific context. Based on two experiments, our study shows that this is actually not the case. In fact, while we observed illusory increases in judged speed with and without sport-specific contextual information, the auditory information only influenced anticipatory judgments regarding the ball’s landing location if the sport-specific context was available. This finding shows that context matters when visual and auditory information influence anticipatory judgments in sports. In addition, it carries the implication that training protocols aimed at improving anticipatory skills are most promising if they include the specific context the training is targeted to prepare for.

## Introduction

Since its infancy, research on anticipatory judgments in sports has predominantly focused on the pick-up of visual information (e.g., Abernethy, 1990; Huys et al., 2009; Ward et al., 2002; for a review, see Loffing & Cañal-Bruland, 2017). Only recently, researchers have started to broaden the scope and examine the impact of other sensory modalities on anticipation such as the processing of auditory information (e.g., Camponogara et al., 2017; Sors et al., 2017). This line of research provides first evidence that auditory information indeed influences visual anticipation in sports such as volleyball (Sors et al., 2017) or racket sports such as tennis (Cañal-Bruland et al., 2018; Müller et al., 2019; Sinnett & Kingstone, 2010). For instance, Cañal-Bruland and colleagues (2018) invited participants to attend to videos of tennis rallies and to predict the landing position of the ball in the opponent’s half. The authors systematically manipulated the intensity of the sounds emanating from racket-ball-contact. They observed that increasingly louder racket-ball-contact sounds systematically biased estimates of ball flight trajectories as well as estimates of how hard the ball had been struck, thus demonstrating an auditory impact on anticipatory judgments. This finding is in line with a previous study by Sors et al. (2017) showing that participants were more accurate in discriminating the speed of volleyball smashes when auditory information compared to visual information regarding the smash-impact was provided. Together, these studies underpin that the auditory modality contributes to judgments of a ball’s speed (Sors et al., 2017) as well as to predictions of its future location (e.g., Cañal-Bruland et al., 2018) in sport-specific contexts.

In the current study, we examined whether context availability (such as the tennis scenario) is a necessary prerequisite for the influence of auditory information on anticipatory judgments. More specifically, we scrutinized whether the impact of auditory information on anticipatory judgments is modulated by the specific context or rather by sound affecting speed judgments independent of context.

Initial evidence from research on visual anticipation shows that context contributes to successful anticipation (for overviews, see Cañal-Bruland & Mann, 2015; Loffing & Cañal-Bruland, 2017; Williams & Jackson, 2019). For instance, Murphy et al. (2016) examined the role of contextual information in visual anticipation by showing skilled and less-skilled tennis players either normal video sequences of tennis rallies or sequences of the same rallies without the postural cues from the players. The removal of postural cues forced participants to predict the final ball bounce location based on contextual information alone. Results indicated that both groups anticipated the ball bounce location more accurately than predicted by chance also in the contextual information alone condition, thereby showing that contextual information contributes to successful anticipatory judgments. In addition, further analyses revealed that in the condition with contextual information alone, skilled participants not only applied different gaze strategies than less skilled players, but they also provided verbal reports showing that they evaluated the contextual information more thoroughly than their less skilled counterparts.

Similarly, a recent study by Goettker et al. (2021) examining the use of contextual cues to guide predictive gaze behaviors in ice hockey spectators provided further support for the crucial role of contextual information in anticipation. More specifically, Goettker et al. showed that spectators employed fundamentally different (predictive) eye movements to anticipate the motion of the puck when full visual information about the hockey context was present vs. absent (i.e., the moving puck alone was presented against a blank background). Together, these studies suggest that at least in visual anticipation information about the context is used and useful to successfully predict the future location of a moving object such as a ball in tennis (Murphy et al., 2016) or a puck in hockey (Goettker et al., 2021).

However, whether such context effects generalize to auditory influences on anticipation remains to be determined. Alternatively, the effects of auditory stimuli on anticipation in sport might be context-independent. Laboratory research has demonstrated striking effects of coinciding auditory information (typically sine-wave tones) on visual perception. For instance, coinciding tones are capable of altering the number of perceived visual events (Shams et al., 2000), affecting the perception of visual correspondence (Meyerhoff & Scholl, 2018; Meyerhoff & Suzuki, 2018; Sekuler et al., 1997) or persistence (Vroomen & de Gelder, 2000), and guide visual attention toward the audio-visually synchronized events (Meyerhoff et al., 2022; van der Burg et al., 2008). With regard to the anticipatory speed judgements in tennis reported in Cañal-Bruland et al. (2018), Meyerhoff et al. (2022) recently showed that one of two moving discs is perceived as being faster than the other when its directional changes coincide with spatially uninformative tones. The authors showed that this illusory increase in perceived speed was caused by the synchronous presentation and perceptual integration of the audio (tone) and the visual (change of direction of the object) stimuli. It follows that the mere synchronous presentation of audio-visual stimuli may cause an illusory increase in perceived speed, thereby potentially also accounting for the reported effects of auditory information on anticipatory judgments in sports. If true, this auditory effect would be context-independent rather than context-dependent as suggested by the literature on visual anticipation (e.g., Murphy et al., 2016).

To test whether the impact of auditory information on anticipation is context-dependent or context-independent, in the current study we ran two separate experiments. In Exp. 1, the sport-specific context was provided, that is, participants watched short video-clips of tennis rallies that were occluded at racket-ball contact. The sounds of the final racket-ball-contact were experimentally manipulated to be either present or absent. In two counterbalanced blocks, participants faced two different tasks: In one block they judged the ball’s speed; in the other block they indicated the ball’s anticipated landing position. In contrast, the sport-specific context was removed in Exp. 2. That is, based on the original footage used in Exp. 1, we rendered new videos that only displayed a moving circle against a blank background (see also Goettker et al., 2021). In all other aspects the design, procedure and set-up mirrored Exp. 1, including the within-subject manipulation of the presence versus absence of the final racket-ball-contact sounds and the counterbalanced blocks for speed judgments and location anticipation judgments.

## Experiment 1

The aim of Exp. 1 was to extend the research on the effect of auditory information on visual anticipation in tennis, that is, when the sport-specific context is provided (Cañal-Bruland et al., 2018). Besides asking participants to predict the ball’s landing location, we also tested whether participants estimated the ball’s speed to be faster when the sound was present (vs. absent; Meyerhoff et al., 2022; Sors et al., 2017).

### Method

#### Participants

Sample size calculation was based on Cañal-Bruland et al. (2018) who reported effects of auditory information on judgments of ball landing position of at least *d* = 0.87. To detect one-tailed effects of such magnitude in a two-group, repeated measures design with a power of 80% necessitated a sample of at least *N* = 10 (analysis run with G*Power; Faul et al., 2007). In line with these requirements, a total of *N* = 24 student participants (gender: 16 male, 7 female, 1 not indicated; age: *M* = 23.08, *SD* = 3.05, range = 18–29) completed the study. 10 out of 24 participants indicated previous tennis experience. Experienced participants reported a mean experience of 9.5 years (SD = 5.89, Range: 5–14 years).^1^ The study was part of a research program that was approved by the Ethics Committee of the Faculty of Social and Behavioral Sciences at the Friedrich Schiller University Jena.

#### Materials & measures

All experimental instructions and stimuli were implemented using standard web technologies (i.e., html + javascript) and presented on a 24″ (1920 × 1080) screen.

##### Videos

**Fig. 1:**
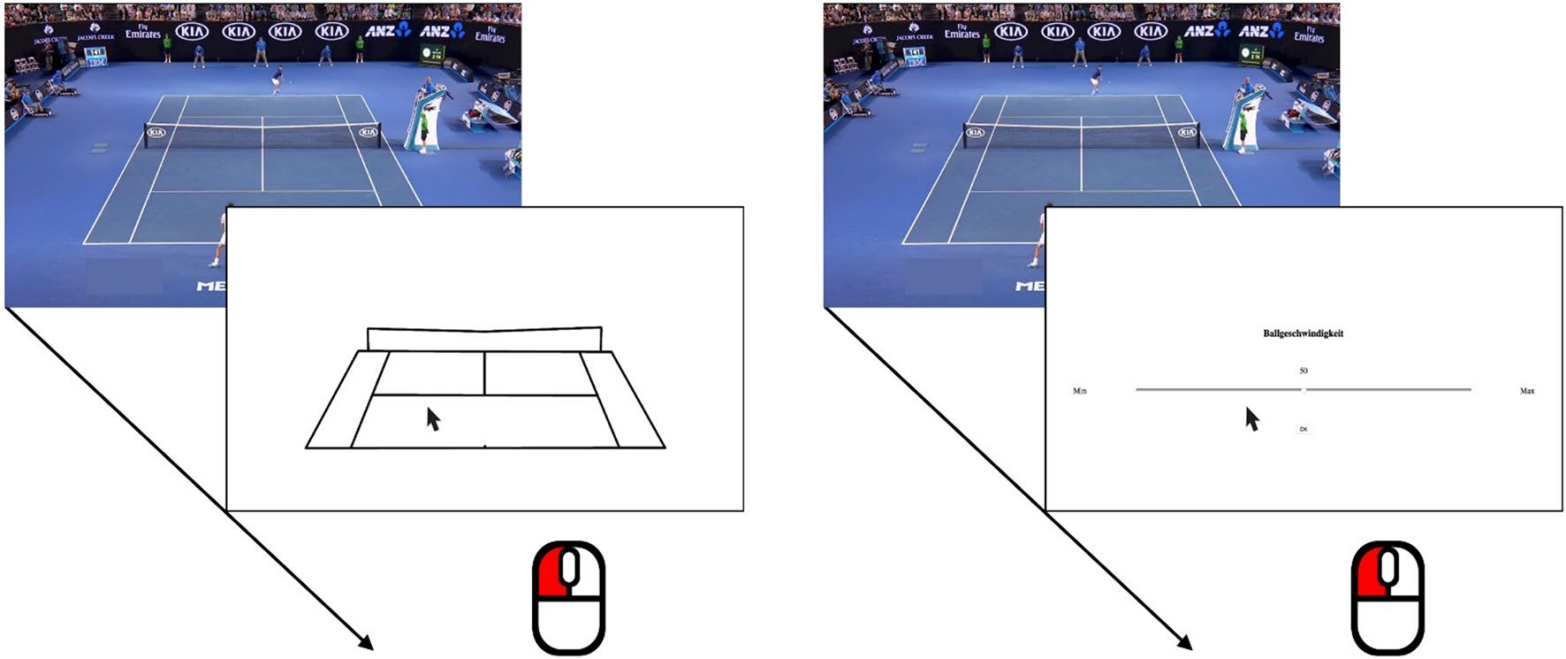
Left panel: In the landing location anticipation block, participants indicated the anticipated landing position of the ball by clicking the location with the mouse. Right panel: In the speed judgment block, participants rated ball speed using an analogue slider (ranging from “min” to “max”; “Ballgeschwindigkeit” is German for “ball speed”) with the mouse

A set of 55 video clips (720*p*, 25*fps*, mean length ≈ 2*s*.) extracted from footage of the semi-final of the Australian Open 2016 between Djokovic and Federer (https://www.youtube.com/watch?v=uEDXMRYe0zo) served as stimuli (taken from Cañal-Bruland et al., 2018). These clips were selected such that they provided the typical raised view of the court, with one player at the top of the screen and the other at the bottom (for an illustration, see Fig. 1). All clips depicted rallies with the same structure, that is the ball was first played by the player at the top, returned by the player at the bottom, and then returned a final time by the player at the top. In order to ensure that participants had to actually anticipate the ball’s trajectory (vs. merely observing), each video was stopped after any racket-ball contact sounds had faded away (mirroring the procedure from Cañal-Bruland et al., 2018). For each video-clip, a version with and without the final racket-ball contact sound was created. This yielded a total set of (55 × 2 =) 110 trials that were shown in individually randomized order. A similar set of three video-clips served as stimuli in the practice trials.

##### Exit questionnaire

The final exit questionnaire entailed questions on demographic information (age, sex), visual impairments (and the use of aids), previous experience with tennis (“Do you have previous experience in tennis?” =  > none, leisure, amateur, professional), and their hypothesis concerning the study (i.e., three open-ended questions on (a) whether they noticed anything unusual, (b) their perceived goal of the study, (c) possible effects of the shown scenarios on anticipation).

#### Procedure & design

Upon arrival at the laboratory, participants first provided informed consent. They were then seated in front of the display which provided all instructions and stimuli on screen. Subsequently, they completed the two experimental blocks. The block order was counterbalanced across participants, constituting the between-subjects factor *Sequence* (1st block: location, 2nd block: speed versus 1st block: speed, 2nd block: location). In the *location anticipation* block, after each video-clip participants had to click on the anticipated landing position of the ball on a sketch of the tennis court (see Fig. 1, left panel). In the *speed judgment* block, participants watched the same video-clips, but rated the speed of the ball by marking their estimate on an analogue slider (see Fig. 1, right panel). Unknown to the participants, each video-clip was shown twice, with the racket-ball-contact sound of the final return either present or absent, constituting the within-subject factor *Sound* (present, absent). To familiarize participants with the block’s task, they were given three practice trials before completing each experimental block. The experiment concluded with the exit questionnaire.

#### Data analysis

In order to test whether the presence vs. absence of racket-ball-contact sounds systematically affects participants’ anticipation of ball landing locations and judgments of speed, both dependent variables were subjected to two separate 2 × 2 repeated measures ANOVAs with *Sound* (present, absent) as within-subject factor and *Sequence* (1st block: location, 2nd block: speed/1st block: speed, 2nd block: location) as between-subject factor. The alpha level for significance was set at 0.05 (one-tailed). As a measure of effect size, we calculated partial eta squared values (*η*_p_^2^).

### Results and discussion

#### Effects of sound on location anticipation

**Fig. 2:**
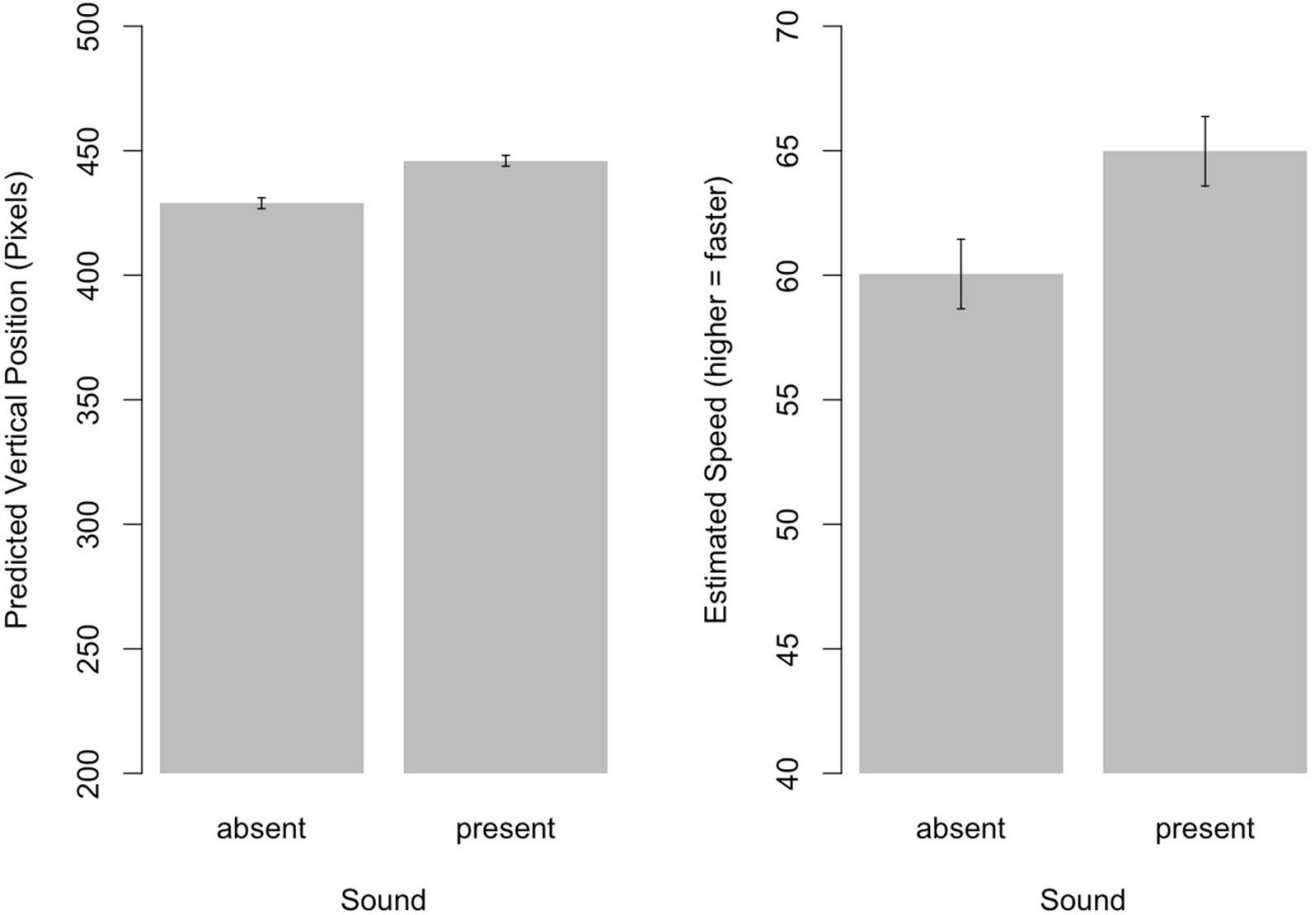
Effects of sound present vs. sound absent on the anticipation judgments of the ball’s landing location in the longitudinal (i.e., vertical) axis (left panel) and on speed judgments (right panel). Higher pixel values indicate longer predicted trajectories, that is, closer to the baseline. Error bars indicate 95% confidence intervals

As the presence of sounds should result in the anticipation of longer trajectories (see Cañal-Bruland et al., 2018; Müller et al., 2019), the vertical component^2^ (*y*-axis value) of the anticipated landing coordinate served as dependent variable (see also Cañal-Bruland et al., 2018; Müller et al., 2019). The 2 (Sound) × 2 (Sequence) ANOVA revealed an effect of *Sound* on the anticipated landing position, *F*(1, 22) = 58.16, *p* < 0.001, *η*_p_^2^ = 0.73, with absent sounds yielding landing positions closer to the net and present sounds yielding landing positions closer to the court baseline (see Fig. 2, left panel). Neither the effect of *Sequence*, *F*(1, 22) = 0.96, *p* = 0.34, nor the interaction *Sound* × *Sequence*, *F*(1, 22) = 0.68, *p* = 0.42, were significant.

#### Effects of sound on speed estimates

Repeating the above 2 (Sound) × 2 (Sequence) ANOVA for participants’ speed ratings again revealed a main effect of *Sound*, *F*(1,22) = 11.88, *p* = 0.002, *η*_p_^2^ = 0.35, with present sounds resulting in higher speed judgments. Again, neither the effect of *Sequence*, *F*(1,22) = 0.11, *p* = 0.75, nor the interaction *Sound *× *Sequence*, *F*(1,22) = 0.79, *p* = 0.38, was significant (see Fig. 2, right panel).^3^

Taken together, the findings confirmed the effects of auditory information on visual anticipation in tennis reported in Cañal-Bruland et al. (2018). That is, when the specific sport context is present, the auditory information systematically affected the anticipation of the landing position of the ball. The results further corroborate that participants estimated the ball’s speed to be faster when the sound was present compared to when it was absent (Cañal-Bruland et al., 2018; Meyerhoff et al., 2022; Sors et al., 2017). To examine whether this effect may occur independent of context information, we conducted Exp. 2.

## Experiment 2

Meyerhoff et al. (2022) recently showed that the synchronous presentation of audio-visual stimuli may cause an illusory increase in perceived speed. It is hence conceivable that such an effect may also drive the established effects of auditory information on anticipatory judgments in sports (e.g., Cañal-Bruland et al., 2018; Müller et al., 2019) including the findings reported in Exp. 1. To test whether the auditory effect on anticipatory judgments emerges independent of context, Exp. 2 closely mirrored the approach detailed in Exp. 1, but differed in one crucial aspect. Instead of presenting participants with stimuli portraying a tennis-specific context, based on the ball trajectory information from each original video used in Exp. 1, we rendered corresponding new abstract (i.e. context-free) video stimuli that displayed a moving circle against a blank background. Instead of the racket-ball-contact sounds, directional changes were accompanied by short beeps. Identical to the manipulation of the within-subjects factor *Sound* (present, absent) in Exp. 1, the final directional change of the ball/circle was either accompanied with a sound (present) or not (absent).

### Methods

#### Participants

In line with Study 1, a similar student sample was collected, that is a total of *N* = 30 participants (gender: 9 male, 18 female, 3 not indicated; age: *M* = 24, *SD* = 4.32, range = 19–36) took part in the study. All experimental procedures were ethically approved by the institutional review board of the Leibniz-Institut für Wissensmedien, Tübingen, and all participants provided informed consent prior to testing.

#### Materials & measures

All experimental instructions and stimuli were implemented using standard web technologies (i.e., html + javascript). The experiment was presented with the Firefox browser on 23″ monitors which were controlled by a MacMini. The participants entered their responses with a standard computer mouse.

##### Videos

**Fig. 3:**
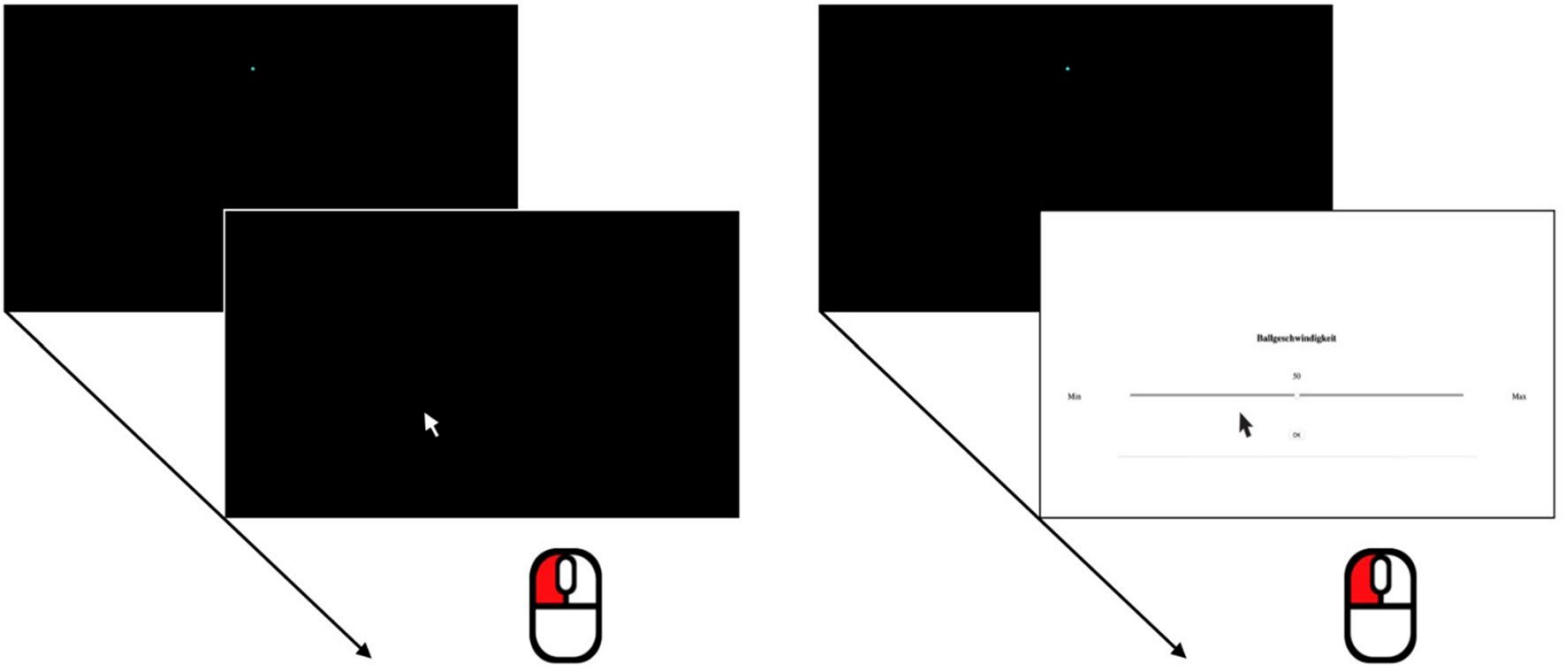
Left panel: In the location anticipation block, participants indicated the position where they anticipated that the observed circle would next change its trajectory. Right panel: In the speed judgment block, participants rated the circle’s speed using an analogue slider (ranging from “min” to “max”) with the mouse

**Fig. 4:**
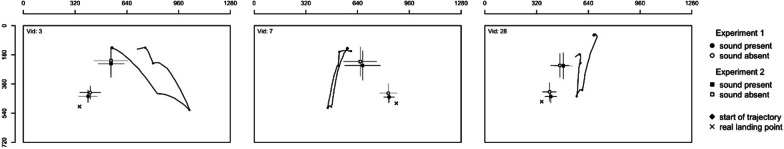
Examples of presented trajectories including real landing position and estimated landing positions by experiment and sound condition. Whiskers indicate standard deviation of participants’ estimates for the respective video

Stimuli were directly derived from the video-clips in Exp. 1. Specifically, using a purpose built website, ball position and direction changes in each video were coded manually frame by frame. The resulting ball position data for each video-clip were then used to render corresponding new videos depicting only a moving turquoise circle against a solid black background (see Fig. 3). We decided to use a black background as this reflects the standard in most visual perception studies. With regard to the ball, we changed the color from yellow to turquoise to avoid that participants generate any associations with the tennis-specific origin of the motion trajectories. In order to provide equally context-free auditory information, each video was combined with a new audio track, containing short sinewave tones (440 Hz, 100 ms, approx. 60 dB, 5 ms on- and offset ramps) coinciding with each direction change. Figure 4 provides representative examples of the circle (ball) trajectories.

##### Exit questionnaire

The final questionnaire was similar to Exp. 1.

#### Procedure & design

Exp. 2 mirrored the procedure and design described in Exp. 1, with the exception of the employed stimuli (see above). In addition, the instruction for the location anticipation task had to be rephrased for the context-free condition. Instead of asking where the ball would bounce and hence next change its trajectory, participants were instructed to indicate where the observed circle would next change its trajectory.

#### Data analysis

Analyses were identical to Exp. 1. That is, both location anticipation judgments and speed ratings were subjected to two separate 2 × 2 repeated measures ANOVAs with *Sound* (present, absent) as within-subject factor and *Sequence* (1st block: location, 2nd block: speed/1st block: speed, 2nd block: location) as between-subject factor.

### Results and discussion

#### Effects of sound on location anticipation

**Fig. 5:**
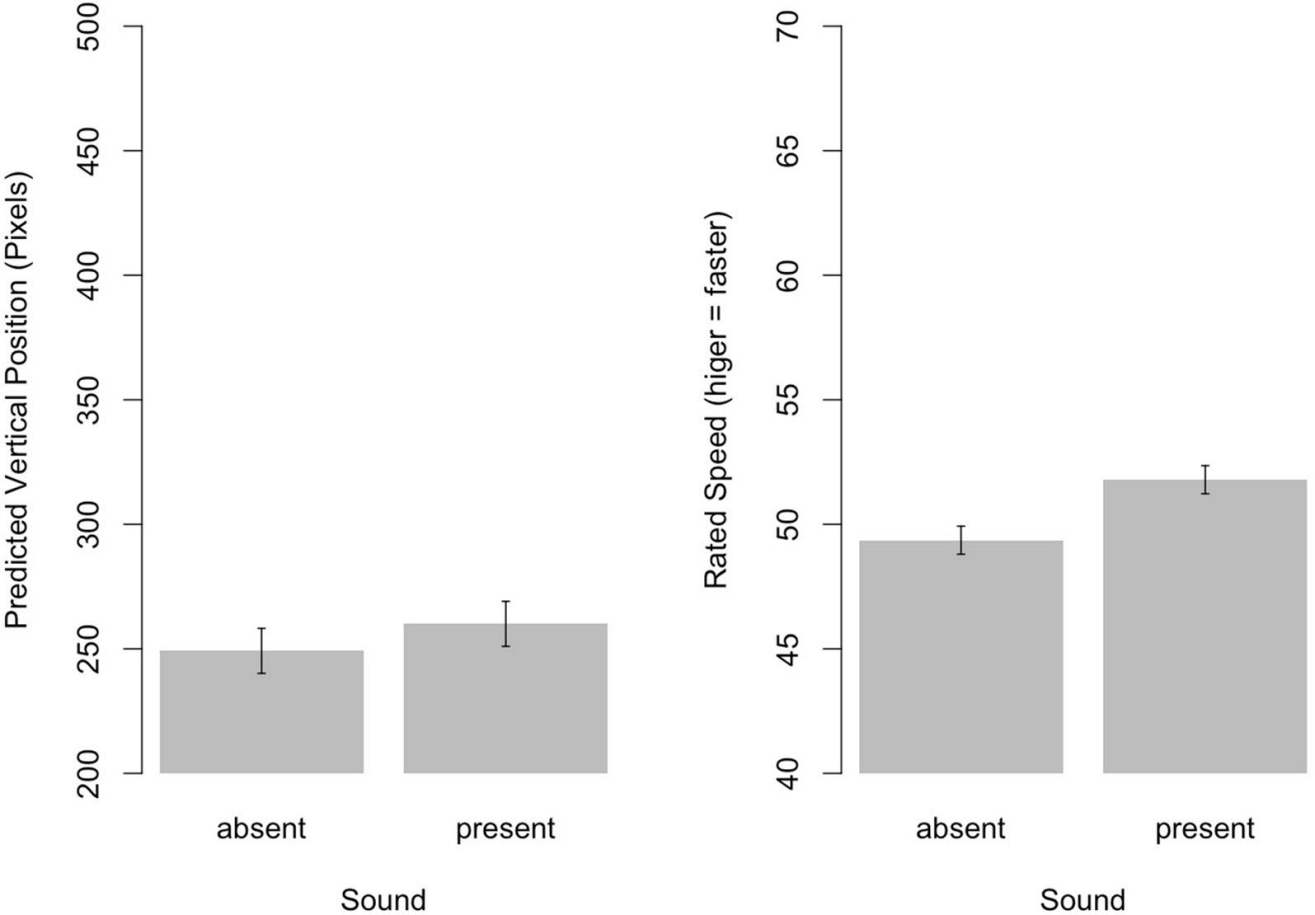
Effects of sound present vs. sound absent on judgments of the anticipated location in the longitudinal (i.e., vertical) axis (left panel) and speed judgments (right panel). Higher pixel values indicate longer predicted trajectories in the direction of the court baseline. Error bars indicate 95% confidence intervals

As the presence of sounds should result in the anticipation of longer trajectories, the vertical component (*y*-axis value) of the anticipated location’s coordinate again served as dependent variable.^4^ In contrast to Exp. 1, however, the 2 (Sound) × 2 (Sequence) ANOVA revealed no main effect for *Sound*, *F*(1,28) = 1.18, *p* = 0.29, *η*_p_^2^ = 0.04. In addition, there was neither a main effect for *Sequence*, *F*(1,28) = 0.82, *p* = 0.37, nor an interaction *Sound *× *Sequence*, *F*(1,28) = 0.15, *p* = 0.70 (see Fig. 5, left panel).

#### Effects of sound on speed ratings

Repeating the 2 (Sound) × 2 (Sequence) ANOVA for participants’ speed ratings revealed a main effect of *Sound*, *F*(1,28) = 16.77, *p* < 0.001, *η*_p_^2^ = 0.37, with present sounds yielding higher speed estimates. Again, neither the factor *Sequence*, *F*(1,28) = 0.28, *p* = 0.60, nor the interaction *Sound* x *Sequence*, *F*(1,28) = 0.30, *p* = 0.59, were significant (see Fig. 5, right panel).

In sum, when no contextual information was provided, participants’ speed judgments were still affected by the sound manipulation (see also Meyerhoff et al., 2022). However, similar to findings from research on visual anticipation (Goettker et al., 2021; Murphy et al., 2016), the removal of context information extinguished the effect of auditory information on participants’ location anticipation judgments. It follows that the impact of auditory information on visual anticipation is context-dependent, while the impact of auditory cues on speed judgments is not. The theoretical implications of these findings will be discussed below.

## General discussion

In this study, we examined whether the impact of auditory information on visual anticipation is dependent or independent of the specific context. Arguments in favor of context-dependency are based on recent research on visual anticipation (e.g., Murphy et al., 2016) and anticipatory gaze behaviors (e.g., Goettker et al., 2021), indicating that contextual information is used to successfully predict the future location of a ball in tennis or a puck in hockey, respectively. Arguments in favor of context-independency stem from research showing that the synchronous presentation of audio-visual stimuli—without any additional context information—may cause an illusory increase in perceived object speed (Meyerhoff et al., 2022), which in turn may account for the reported effects of auditory information on anticipatory judgments in sports (e.g., Cañal-Bruland et al., 2018; Müller et al., 2019).

To test these competing hypotheses, we invited participants to watch video snippets of tennis rallies in which racket-ball-contact sounds were either present or absent in Exp. 1, and in which they watched the same trajectories without any specific context information (i.e., videos displayed only a moving circle against a blank background) in Exp. 2. In both experiments, participants judged the anticipated bounce location of the ball/circle and its speed in separate blocks. Results of Exp. 1 showed that when the sport-specific context was provided, the presence (vs. absence) of racket-ball-contact sounds resulted in longer estimates of the ball flight trajectory as well as faster speed judgments, thereby both confirming and extending earlier findings (e.g., Cañal-Bruland et al., 2018; Müller et al., 2019; Sors et al., 2017). Results of Exp. 2, on the one hand, showed that even in the absence of contextual information the presence (vs. absence) of contact sounds continued to result in faster speed estimates. This result confirms recent findings showing that the synchronous presentation and perceptual integration of the audio (tone) and visual (change of direction of the object) stimuli causes illusory increases in increased speed (Meyerhoff et al., 2022). On the other hand, however, this increase of speed estimates was not accompanied by an effect on location anticipation. We interpret this finding such that perceptual effects of audio-visual integration (e.g., illusory increases in perceived speed) can be dissociated from implied consequences such as longer motion trajectories of faster objects. This interpretation is supported by additional analyses,^5^ showing no correlations between location anticipation and speed estimates, further corroborating that these judgments are likely based on different processes. An additional suggestion for future research to examine these processes would be to re-run this task, but employ a different dependent variable, namely temporal estimates of when participants think the ball would land or the object would change direction following racket/ball contact (for example, see Schroeger et al., 2022).

Regardless, while illusory percepts emerge independent of contextual information, the anticipation of landing positions seems to require the presence of contextual information such as the tennis court and/or relational information between the players and the ball (Murphy et al., 2018). In this context, it should be noted that in our study we opted to either present the entire context including the reference frame (i.e. court etc.), player movements, relational information between players and the ball etc. (Exp. 1) or remove all of this contextual information (Exp. 2). While our study clearly shows an impact of contextual information, more research is needed to examine whether the presentation of isolated parts of the contextual information (e.g., the reference frame only) may be sufficient to elicit the effects reported in our manuscript.

It follows that, together, the results of Exp. 1 and Exp. 2 provide evidence against a pure context-independent effect of auditory information on location anticipation, but—similar to findings regarding the impact of contextual information on visual anticipation (see e.g., Goettker et al., 2021; Murphy et al., 2016)—in favor of a context-dependent effect of auditory information on anticipation (Cañal-Bruland et al., 2018; Müller et al., 2019; Sors et al., 2017).

These findings carry a number of practical implications. First, they provide further evidence that not only visual information, but also auditory information plays a crucial role in anticipation in sports (e.g., Camponogara et al., 2017; Cañal-Bruland et al., 2018; Müller et al., 2019; Sinnett & Kingstone, 2010; Sors et al., 2017). Hence, to improve training protocols and performance the multiple sensory channels should be considered and trained. Second, similar to visual information (for overviews, see Cañal-Bruland & Mann, 2015; Loffing & Cañal-Bruland, 2017), the current study shows that the impact of auditory information on anticipation is context-dependent. It follows that training protocols aimed at the improvement of anticipatory skills and the integration of multisensory information are most promising if they include the specific-context the training is targeted to prepare for (for similar arguments, see Williams & Jackson, 2019). Future research is needed, on the one hand, to test these recommendations for the improvement and training of anticipation and, on the other hand, to explore to what degree these implications generalize to other domains.

## Data Availability

The data will be shared in an open repository such as Harvard Dataverse upon acceptance for publication.

## Footnotes

1 To check whether experience may have affected our results, we re-ran all analyses reported in the results section for Exp. 1, only including the inexperienced participants. Results confirmed that concerning all ANOVA’s (i.e., for anticipated location, speed judgments, and radial errors) results were identical to the full sample, that is, only main effects of Sound (present, absent) were observed (all *p*’s < .02).

2 For the sake of completeness, horizontal landing positions were subjected to the same analyses. Only an interaction of Sound × Sequence (*p* = .033) was found. Post hoc analyses using pairwise comparisons between both sound conditions, separately for each sequence, as well as between both sequence conditions, separately for both sound conditions, revealed no pairwise differences (all *p*’s > .09). Analyses of radial errors mirrored those for *y*-position: Only an effect of Sound, F(1, 22) = 6.51, p = .02, *η*_p_^2^ = .23, was found (all other *p*’s > .46).

3 Note that the follow-up questions revealed that a subset of participants (*N* = 3) guessed the study's hypothesis. We did not remove those participants from the reported analyses, as repeating the same analyses without these participants yielded virtually identical results.

4 No effects were found for horizontal landing positions (all *p*’s > .22). Repeating the analysis for the radial error between the estimated and real landing positions for each trial mirrored the analysis on y-position, that is, none of the effects were significant (all *p*’s > .19).

5 For Experiment 1, after removal of one bivariate outlier with more than 2 SDs from the mean for both variables. there was a non-significant correlation between anticipation location judgments and speed estimates of *r* = .35, *p* = .10. Similar to Exp. 1, for Exp. 2 after removal of an extreme outlier, analysis yielded a non-significant correlation between anticipation location judgments and speed estimates of *r* = −.16 (*p* = .40). We also checked whether these bivariate outliers would in any way affect the findings from the analyses reported in the results section. With the exception of the interaction effect of *Sound *× *Sequence* on predicted *x*-positions in Experiment 1 (see footnote 2; which after removal was no longer significant) all other effects remained unchanged.
